# Effectiveness of computerized clinical decision support systems for asthma and chronic obstructive pulmonary disease in primary care: a systematic review

**DOI:** 10.1186/1471-2466-14-189

**Published:** 2014-12-02

**Authors:** Mariam Fathima, David Peiris, Pradnya Naik-Panvelkar, Bandana Saini, Carol Lyn Armour

**Affiliations:** Sydney Medical School, University of Sydney, Sydney, New South Wales Australia; Woolcock Institute of Medical Research, Sydney Medical School, University of Sydney, Sydney, New South Wales Australia; The George Institute for Global Health, Sydney Medical School, University of Sydney, Sydney, New South Wales Australia; Faculty of Pharmacy, University of Sydney, Sydney, New South Wales Australia

**Keywords:** CCDSS, Computerized clinical decision support systems, Asthma, COPD, Computerized clinical decision making, Systematic review

## Abstract

**Background:**

The use of computerized clinical decision support systems may improve the diagnosis and ongoing management of chronic diseases, which requires recurrent visits to multiple health professionals, disease and medication monitoring and modification of patient behavior. The aim of this review was to systematically review randomized controlled trials evaluating the effectiveness of computerized clinical decision systems (CCDSS) in the care of people with asthma and COPD.

**Methods:**

Randomized controlled trials published between 2003 and 2013 were searched using multiple electronic databases Medline, EMBASE, CINAHL, IPA, Informit, PsychINFO, Compendex, and Cochrane Clinical Controlled Trials Register databases. To be included, RCTs had to evaluate the role of the CCDSSs for asthma and/or COPD in primary care.

**Results:**

Nineteen studies representing 16 RCTs met our inclusion criteria. The majority of the trials were conducted in patients with asthma. Study quality was generally high. Meta-analysis was not conducted because of methodological and clinical heterogeneity. The use of CCDSS improved asthma and COPD care in 14 of the 19 studies reviewed (74%). Nine of the nineteen studies showed statistically significant (p < 0.05) improvement in the primary outcomes measured. The majority of the studies evaluated health care process measures as their primary outcomes (10/19).

**Conclusion:**

Evidence supports the effectiveness of CCDSS in the care of people with asthma. However there is very little information of its use in COPD care. Although there is considerable improvement in the health care process measures and clinical outcomes through the use of CCDSSs, its effects on user workload and efficiency, safety, costs of care, provider and patient satisfaction remain understudied.

**Electronic supplementary material:**

The online version of this article (doi:10.1186/1471-2466-14-189) contains supplementary material, which is available to authorized users.

## Background

Chronic respiratory diseases, particularly asthma and chronic obstructive pulmonary disease (COPD), kill more than four million people every year world-wide and affect hundreds of millions more [1]. Around 300 million people suffer from asthma world-wide, with a projected increase of an additional 100 million people by 2025 [1]. The economic burden of asthma has been estimated to be the highest among chronic diseases [2] and includes both direct (e.g. hospital admissions and costs of medications) and indirect costs (e.g. days away from work) [2, 3]. The Global Burden of Disease Study projected that COPD, which ranked sixth as a cause of death in 1990, will become the third leading cause of death in 2030 [4].

Effective management of chronic diseases requires optimal dissemination and implementation of guidelines, however there is a gap between scientific evidence-based medicine and real clinical practice, especially in primary care [5]. Although effective therapies and guidelines are available, many patients with asthma still have frequent, uncontrolled symptoms and do not receive optimal care. Research demonstrates that only a quarter of patients with persistent asthma symptoms take anti-inflammatory medications as recommended by the guidelines [6]. Much of the cost of asthma care is attributable to poor disease control due to non-adherence to guideline-recommended controller therapies [7, 8], over reliance on reliever medication [9], inadequate monitoring of disease severity and insufficient patient education for effective self-management [10].

Similarly, the care provided for patients with COPD in community settings indicates low level of awareness and implementation of guidelines [11–13], despite the high level of evidence for the efficacy of guideline-based interventions. Medication use is often not in accordance with the guidelines [14] and a high proportion of patients prescribed with inhalers use them incorrectly [15, 16]. Smoking cessation can reduce the rate of decline in lung function, yet many with COPD continue to smoke. Influenza and pneumococcal vaccinations can reduce the rate of exacerbations, hospitalizations and death [12]. However, in Australia, for example, based on the 2004-05 National Health Survey 25% and 59% of those with self-reported COPD had never been administered influenza or pneumococcal vaccinations respectively [3].

Globally, studies evaluating the provision of care by clinicians suggest that evidence-based care was delivered approximately 40-55% of the time [17–19]. The reasons for sub-optimal uptake of guidelines into practice are complex and occur at the patient, provider and system levels [20]. Given, the rising global disease burden from asthma and COPD and intractable health system deficits in providing evidence based care there is a pressing need to identify systems-focused solutions. Computerized Clinical Decision Support System (CCDSS) is well established as one strategic method of improving care for prevention and management of chronic conditions. A CCDSS is “any electronic information system based on a software algorithm designed to aid directly in clinical decision making, in which characteristics of individual patients are used to generate patient-specific assessments or recommendations that are then presented to clinicians for consideration” [21]. CCDSS is valuable not only to the clinicians, but can also provide other health care providers, patients, or caregivers with clinical knowledge and patient-specific information to help them make decisions that enhance patient care [22]. Typically CCDSS interventions include forms and templates for entering and documenting patient information, and alerts, reminders, and order sets for providing suggestions and other support.

Importantly, CCDSS interventions can increase adherence to evidence-based medical knowledge, reduce unnecessary variation in clinical practice and improve their clinical decision-making process [21, 23].

CCDS systems that are well designed and implemented have the potential to improve health care quality, increase efficiency by reducing mental workload, improve clinical work-flow and reduce health care costs [23, 24]. CCDSS has been used in the management of various chronic conditions such as diabetes [25, 26], hypertension [27, 28], dyslipidemia [29, 30] and cardiac care [31] across various health care settings.

Although there have been reviews of the effectiveness of CCDSSs in the management of various disease states in different clinical settings [32, 33], there have been no systematic appraisals of their impact on chronic respiratory diseases such as asthma and COPD in primary care. Our systematic review aimed to synthesize evidence for its use in the care of patients with asthma and COPD and to identify the key features of those systems that have the potential to overcome health system barriers and improve outcomes.

## Methods

### CCDSS definition

CCDSS was defined as an automated process for comparing patient-specific characteristics against a computerized knowledge base with resulting recommendations or reminders presented to the provider (or the user) to consider, to help them in clinical decision making.

### Search strategy

The electronic databases MEDLINE, EMBASE, CINAHL, IPA, Informit, PsychINFO, Compendex, and Cochrane Clinical Controlled Trials Register databases were reviewed by the primary author. MeSH terms ‘clinical decision support systems/tools/techniques/aids/guidelines’, ‘computer assisted therapy/diagnosis/decision making’, ‘computeris(z)ed decision making’, ‘CCDSS’, ‘medical informatics’, ‘asthma’, ‘COPD’ and combinations thereof were included. The detailed search strategy that was used in MEDLINE is outlined in Table 1. This search strategy was repeated in all other databases. We also systematically searched the reference lists of all the included studies and relevant reviews.

**Table 1:** **Search strategy for Medline**

	Search terms used
*1.*	*exp Decision Support Systems, Clinical/*
*2.*	*clinical decision support systems.tw.*
*3.*	clinical decision support tool*.tw.
*4.*	clinical decision support system*.tw.
*5.*	*Decision Support Techniques/*
*6.*	*Medical Order Entry Systems/*
*7.*	*Decision Making, Computer-Assisted/*
*8.*	*Diagnosis, Computer-Assisted/*
*9.*	clinical decision support aid*.tw.
*10.*	clinical decision support guideline*.tw.
*11.*	*computer assisted therap$.tw.*
*12.*	*Therapy, Computer-Assisted/*
*13.*	reminder system*.tw.
*14.*	*Reminder Systems/*
*15.*	*computeri?ed clinical decision support.tw.*
*16.*	*CCDS.tw.*
*17.*	medical informatic*.tw.
*18.*	*Medical Informatics/*
*19.*	*1 or 2 or 3 or 4 or 5 or 6 or 7 or 8 or 9 or 10 or 11 or 12 or 13 or 14 or 15 or 16 or 17 or 18*
*20.*	*asthma.mp. or exp Asthma/*
*21.*	*exp Pulmonary Disease, Chronic Obstructive/*
*22.*	*chronic obstructive pulmonary disease.tw.*
*23.*	*COPD.tw.*
*24.*	*20 or 21 or 22 or 23*
	*19 and 24*
*25.*	*19 and 24*
*26.*	*limit 25 to (English language and humans)*
*27.*	*limit 26 to yr = ”2000 -Current”*

The asterisk (*) represents any group of characters, including no character. The question mark (?) represents any single character. The dollar sign ($) represents zero or one character (used when searching for expressions).

#### Inclusion criteria

-  Empirical studies published in the English language between 2003 to May 2013;
-  Pediatric or adult CCDSS interventions involving COPD and/or asthma screening, prevention, case detection, and management;
-  Randomized controlled trials comparing CCDSS with explicitly defined clinical or process outcome measures;
-  CCDS system used by any clinicians (physicians, physician assistants, pharmacists, dentists, pulmonary specialists or nurse practitioners) directly involved in patient care;
-  CCDSS targeting patients in improving self-management.

#### Exclusion criteria

-  Review articles, conference proceedings, meeting abstracts;
-  Paper-based tools (e.g. flow charts and non-electronic clinical pathway tools);
-  CCDSS interventions in people with other conditions (rather than asthma and COPD), including other respiratory diseases;
-  CCDSS for medical education purposes or only providing summaries of information for patients;
-  Group based interventions that did not include individual clinical assessment;
-  Evaluations which focused only on the technical performance of the system as opposed to its effect on clinical practice;
-  In-patient hospital based systems.

### Study selection

Two authors (MF and PNP) independently reviewed the titles, index terms, and abstracts of the identified references and rated each paper as “potentially relevant” or “not relevant” based on study design, subjects, setting, and intervention. These two authors then independently reviewed the full texts of the selected potentially relevant articles and again rated each paper as “potentially relevant” or “not relevant”. After application of the full set of inclusion and exclusion criteria to the potentially relevant studies, a further limitation was then applied, and only RCTs were included. Disagreements between reviewers were resolved by discussion with a third author (CA) until consensus was reached.

### Data extraction and quality assessment

The primary author (MF) independently extracted data related to Participants, Intervention, Comparator, Outcomes and Study design by utilizing the PICOS strategy for describing trials (Table 2). The second author (PNP) then independently examined the studies and extracted data to confirm accuracy. The data abstracted included the following information: manuscript authors, year of publication, the study design and duration, participant characteristics (health practitioners, patients), the type of CCDSS intervention, the comparator (usual care or another form of CDSS) and the outcomes measured (clinical, process, workload and efficiency, economic and implementation). Bias was assessed using the Cochrane risk of bias tool [34], and was based on the following five dimensions: randomization, allocation concealment, blinding of participants, personnel or outcome assessors, selective outcome reporting and completeness of follow-up [duration of follow-up, intention to treat (ITT) analysis, withdrawals, and reasons for dropouts]. Each of the above attributes was assessed as being high, low or unclear and an overall risk of bias was reached for each of the included studies (Table 3).

**Table 2:** **Design and characteristics of the included studies**

Citation, year, country	Population: no. of centers/providers/patients (Intervention, I or Control, C)	Study design, setting and duration	Intervention	Comparator	Outcome measures	Key findings and effect size	Comments
1. Carroll et al. [35] USA.	1/-/2098 (children aged 3-11 years) (I = 1082, C = 1016)	RCT, Community based. Duration: 21 months	Parent survey on the presence of asthma symptoms linked sequentially to physician prompts. Physician prompts mediated by CDSS.	Parents received no screening questions, and physicians received no prompts.	**Primary outcome**: Physicians’ diagnosis of childhood asthma based on prompts by the CDSS.	(+) effect. The number of children diagnosed with asthma in the intervention group was significantly more compared to the control group (8.6% vs. 5.8%, P <0.02). Effect size Cohen’s d = 0.24, 95% C.I = (0.04-0.43)	Not clear if physician training was provided.
2. Hashimoto et al. [36] Netherlands	6/-/95 (adults with adults diagnosed with severe asthma) (I = 51, C = 38)	Pragmatic Multicentre RCT, Academic and community setting Duration: 6 months	Internet-based management tool involving home monitoring of symptoms (using an electronic diary), treatment decision support for the patients, and monitoring support by a study nurse.	Conventional asthma treatment by pulmonologists	**Primary outcome:** Cumulative sparing of oral corticosteroids, asthma control using Asthma control Questionnaire (ACQ), asthma-related quality of life (AQLQ), **Secondary outcomes:** FEV1 (using Piko-1 device), exacerbations, hospitalizations and satisfaction (Global satisfaction scale)	(+) effect. Median cumulative sparing of prednisone was 205 mg in the internet group compared with 0 mg in the conventional group. (P = 0.02) Asthma control, AQLQ, FEV1, exacerbations, hospitalizations and satisfaction with the strategy were not different between groups. Effect size for CCS sparing effect: Cohen’s d = 0.46. 95% C.I = (0.08- 0.93)	Patients were trained in using the electronic self -management support system, recording symptoms, measuring lung function and fraction of exhaled nitric oxide (FENO)
3. Van der Meer et al. [37], Netherlands	37 general practices and 1 academic outpatient department/69/200 (adults with asthma) (I = 101, C = 99)	Multi-centre, RCT, Community and Academic setting. Duration = 12 months	Internet-based asthma self-management program consisting of weekly asthma control monitoring and treatment advice, online support and group education delivered via remote web communications by a specialized asthma nurse	Usual physician-provided asthma care	Process outcomes: (asthma knowledge, inhaler technique and self-reported medication adherence), health care provider contacts for asthma, use of internet based asthma monitoring tool, and medication changes. Clinical outcomes: **Primary:** Asthma-related quality of life, (32-item Asthma Quality of Life Questionnaire). Secondary: Asthma control (ACQ), symptom-free days, pre-bronchodilator FEV1 (Piko-1), daily inhaled corticosteroid dose, and exacerbations	(+ but modest effect) Improvement in asthma knowledge, inhaler technique and slightly fewer physician visits in the internet group. Treatment changes occurred more often in the internet group. Modest improvement in asthma control and lung function with the Internet intervention, but no reduction in exacerbations. Improvement in asthma-related quality of life was slightly less than clinically significant. (P ≥ 0.5). Effect size for the primary clinical outcome (AQLQ increase by >0.5): Cohen’s d = 0.6. 95% C.I = (0.3-0.9)	Education and training provided to the participants. Non-blinded nature of the study may have affected the results
4. Van der Meer et al. [38] Netherlands	37/69/200 (adults with partly controlled or uncontrolled asthma) (I = 101, C = 99)	Prospective RCT, Community and academic. Duration: 1 year	Weekly internet based self-monitoring (using ACQ) and subsequent treatment adjustment (using an online management algorithm)	Usual care by the general practitioner according to the Dutch GP guidelines based on GINA guidelines	**Primary:** Asthma control using (ACQ), spirometry and ATAQ (asthma therapy assessment questionnaire).	(+) effect. Significant improvements in ACQ score after 12 months in the internet based self-monitoring group. Daily inhaled corticosteroid dose significantly increased in the Internet group compared to usual care in the first 3 months in patients with uncontrolled asthma, but not in patients with well or partly controlled asthma. After one year there were no differences in daily inhaled corticosteroid use or long-acting β2-agonists between the Internet group and usual care.	Patients were trained to measure (FEV1) daily with a hand-held electronic spirometer (PiKo1). Supervision provided by a nurse specialist. So there is heavy initial investment. Study outcomes were self-reported by the patients which may overestimate effect. The effect size for change in asthma control is quite large, esp. in the uncontrolled group making the intervention promising.
					**Secondary:** Mean daily dose of inhaled corticosteroid (ICS), and the proportion of participant’s using long-acting β2-agonists (LABA) or leukotriene receptor antagonists (LTRA).	**Effect size primary outcome** i.e. change in ACQ in partly controlled asthma group*: Cohen’s d = 0.81 95% C.I = (0.33- 1.35)	
						**Effect size for change in** ACQ in uncontrolled asthma group*: Cohen’s d = 0.94. 95% C.I = (0.38-1.5). *Assuming *t*-test was performed.	
5. Taylor et al. [39] Australia.	3/50/1 (simulated patient) (I = 27, C = 23 ED doctors)	RCT, Community setting, Duration: 4 months	An integrated and dynamic electronic decision support system for management of acute asthma in the emergency department (ED) by ED physicians.	Acute asthma management using paper -based clinical records, treatment order sheets and discharge documentation.	Work load & efficiency outcomes: **Primary:** Quality of asthma documentation–measured using 10 documentation variables (clinical parameters and discharge documentation). Secondary: consultation time	(+) effect. Significantly higher rates of documentation in 7 out of 10 variables, including provision of written short-term asthma management plans. No significant difference in consultation times. **Effect size for documentation of asthma management plan** provision: Cohen’s d = 0.78. 95% C.I = (0.18-1.37)	Relevant to the ED setting. A 2 minute introduction to the system, including basic functions of the program provided to physicians, which may not be enough. One simulated patient case may not reflect a spectrum of scenarios faced in the ED setting
6. Fiks et al. [40] USA.	20/-/11919 (children with asthma between 5-19 years of age) (I = 6110, C = 5809)	Cluster-RCT, Academic. Duration: 5 months.	Electronic health record (EHR) based influenza vaccine clinical alerts	Routine care	Health care process outcomes- rates of captured opportunities for influenza vaccination (visit-level analysis) and up-to-date influenza vaccination status among patients with asthma	(+) but modest effect. Standardized influenza vaccination rates improved 3.4% more at intervention sites than at control sites. Effect was not significant, Cohen’s d not calculated)	Primary care sites were linked to a teaching hospital. Information on the comparator was unclear-implied usual practice. Training provided to the physicians was quite thorough.
7. Bell et al. [41] USA.	12/-/19450 (children) (I = 6, C = 6) Children with persistent asthma identified by using the pediatric asthma control test (PACT))	RCT, Academic setting. Duration: 1 year	CDSS embedded in an electronic health record (EHR), where it provides support in the management of children with asthma in accordance with the (National Asthma Education Prevention Program guidelines (NAEPP).	Passive asthma management tools available in the electronic health record (EHR).	Health care process outcomes: Proportion of children with at least 1 prescription for controller medication, an up-to-date asthma care plan, and documentation of performed office-based spirometry.	(+) effect. Significant improvement in adherence to NAEPP guidelines. 6% increase in the number of prescriptions for controller medications, (P = 0.006) and 3% increase for spirometry (P = 0.04) in the intervention urban practices. Filing an up-to date asthma care plan improved 14% (P = 0.03) and spirometry improved 6% (P = 0.003) in the suburban practices with the intervention. The effect size could not be calculated, as data provided was insufficient to calculate d values).	Medical practices within the Children’s Hospital of Philadelphia (CHOP) Pediatric Research Consortium-may not be generalizable. Physicians were trained to use the CDSS. The actual number of providers involved in the study is unclear.
8. Rasmussen et al. [42] Denmark.	-/-/300 (adults with asthma)	Multi-centre RCT with three parallel groups. Community setting Duration: 6 months	Physician-managed online interactive asthma monitoring tool which comprised of (1) an electronic diary, (2) an action plan for the patients and (3) a decision support system for the physician. Patients with persistent asthma received advice on treatment based on their asthma control.	Two other usual care groups: specialist group, where treatment was provided by an asthma specialist in an outpatient clinic; and a general practitioner (GP) group, where treatment was provided by GPs in primary care.	**Clinical outcomes** Asthma symptoms: electronic diary. Asthma quality of life: AQLQ) questionnaire. Lung function: Spirometry Airway responsiveness: Methacholine challenge test.	(+) effect. Significant improvement in the Internet group compared to the other 2 groups regarding asthma symptoms, quality of life, lung function, airway responsiveness. Significant improvement in the use of inhaled corticosteroids in the internet and specialist group. Effect size comparing the Internet vs. Specialist group for asthma symptom reduction was Cohen’s d =0.53. 95% C.I = (0.19-0.87). Cohen’s d comparing the Internet vs. GP group for asthma symptom reduction was 0.64. 95% C.I = (0.29-0.99).	The number of practitioners and the number of centers participating were unclear. No training provided to the participants or the GP’s but the laboratory assistants providing spirometry and methacholine test were trained in the required protocol
9. Dexheimer et al. [43] USA.	1/-/704 (Children 2-18 years of age), (I = 358, C = 346)	RCT, Community setting, Duration: 3 months	A fully computerized asthma detection system which printed a paper-based asthma care protocol in the pediatric ED to guide early asthma treatment and reduce time to disposition decision.	Usual care, i.e., no reminders or automatic printout was provided.	**Primary outcome:** Time from ED triage to disposition (discharge or hospital admission) decision.	No effect. No difference in time to disposition. Length of ED stay and the rate of hospital admission were similar between the two groups. (Effect was not significant, Cohen’s d not calculated)	The number of physicians, respiratory therapists and nurses involved in the study is unclear.
					**Secondary outcomes**: Guideline adherence measures including asthma education ordered, protocol found on chart, any asthma scoring performed.		
10. Smith et al. [44] UK.	29/-/911 (patients 5+ years of age with severe asthma) (I = 457, C = 454)	Cluster RCT, Community setting. Duration: 2.5 years	Addition of electronic alerts to computerized records to identify at-risk asthma patients experiencing an exacerbation and modify their care.	Control practices continued usual care.	**Primary outcome:** number of patients experiencing a moderate-severe exacerbation	No significant difference between groups in number of people experiencing exacerbations. Relative reductions in people experiencing hospitalizations, accident and emergency, out-of hours contacts and increase in prednisolone prescriptions for exacerbations without increasing costs. (Effect was not significant, Cohen’s d not calculated)	Training on using electronic alerts provided to at least one representative from each staff group (GP, nurse, receptionist, manager/administrator, dispenser) of the intervention practices.
					**Secondary outcome:** outpatient attendances for asthma, primary care contacts, ‘did not attends’ (DNAs) at consultations, asthma medications and cost analyses		
11. Kattan et al. [45] USA.	-/435/937 (5-11year old with moderate to severe asthma) (I = 471, C = 468)	RCT, Community setting. Duration: 1 year	Computer generated letters based on information collected from the child’s carer through bi-monthly telephone calls conducted by the centralized service for all the study sites. The letter to the physician caring for that child summarized the child’s asthma symptoms, health service use, and medication use with a corresponding recommendation to step up or step down medications in accordance with the NAEPP guidelines.	No letters sent to the providers of the children in the control group	Health care process outcomes: scheduled visits and changes in medications. Patient outcomes: maximum number of symptom days, ED visits and hospitalizations for asthma, and school days missed because of asthma.	(+) effect Significant increase in scheduled visits, (17.1% vs12.3%, P = 0.005). Significant increase in medication step up (46% vs 35.6%, P = 0.03). Significantly fewer ED visits in the intervention group compared with controls (0.87 vs 1.14 per year, p = 0.013). No difference in the maximum number of symptom days and number of school days missed. Effect size for % of scheduled visits resulting in step-up of medication: Cohen’s d = 0.23. 95% C.I = (0.02-0.43)	Intervention practitioners were trained. Effect size was low for medication change related outcomes. Key issues also included the design where not all children whose medication change was warranted visited the physician.
12. Tierney et al. [46] USA.	4/266/706 (246 physicians and 20 outpatient pharmacists)	RCT, Academic setting, Duration: 3 years	Computerized care suggestions to improve asthma and COPD management. These focused on: (1) pulmonary function tests, (2) influenza and pneumococcal vaccinations, (3) prescribing inhaled steroid preparations in patients with frequent symptoms of dyspnea, (4) prescribing inhaled anticholinergic agents in patients with COPD, (5) escalating doses of inhaled β-agonists for all patients with persistent symptoms, (6) prescribing theophylline for patients with COPD and continued symptoms despite aggressive use of inhaled anticholinergic agents, b-agonists, and steroids, and (7) encouraging smoking cessation.	Four groups: physician intervention only, pharmacist intervention only, both pharmacist and physician interventions, and no intervention (controls).	**Primary:** Adherence to guideline based care suggestions.	No effect. No differences between groups in adherence to the care suggestions, quality of life, patients satisfaction with physicians’ or pharmacists, medication compliance, emergency department visits, or hospitalizations. Physicians receiving the intervention had significantly higher total health care costs. Physician attitudes toward guidelines were mixed. (Effect was not significant, Cohen’s d not calculated)	Hospital based academic practices. Providers included internal medicine physicians, residents and pharmacists. Training was provided to the providers. Questionnaires were administered via telephone.
					**Secondary:** Quality of life-McMaster Chronic Respiratory Disease Questionnaire (CRQ) for COPD patients or the McMaster Asthma Quality-of-Life. Questionnaire (AQLQ). Patient satisfaction: American Board of Internal Medicine’s patient satisfaction questionnaire. Medication adherence: Inui and Morisky surveys and pharmacy dispensing records		
13. Martens et al. [47] Netherlands.	-/53/-	Clustered RCT, Community setting Duration: 1 year	A decision support system with reactive computer reminders (CRS) to improve drug prescribing behaviors. 25 GPs received reminders on antibiotics and asthma/COPD prescriptions.	28 GPs received reactive computer reminders (CRS) to improve prescribing of cholesterol-lowering drugs	**Primary outcome:** prescription according to the guidelines as a percentage of total prescriptions of a certain drug. Secondary outcome: user friendliness.	(+) but not significant effect. CRS with reactive reminders improved drug prescribing behavior. Preliminary results also indicate reduction in the number of prescriptions according to the advice of the computerized guidelines not to present certain drugs. It was perceived stable and user friendly. (Effect was not significant, Cohen’s d not calculated)	Preliminary study. Both groups served as control to one another. Not specific to asthma/COPD.
14. Martens et al. [48] Netherlands. Follow-up of the above study	14/53/-	Clustered RCT, Community setting. Duration: 1 year	CRS focused on drug-prescribing behavior of GPs. 25 GPs received reminders on antibiotics and asthma/COPD prescriptions	28 GPs received CRS reminders on cholesterol prescriptions	Guideline appropriate prescriptions as a percentage of total prescriptions (of the drug category involved) for the same diagnosis on the individual GP level. Absolute number of prescriptions for a specific diagnosis per GP per 1000 enlisted patients.	No effect. No favorable effects were found for CRS with the message to prescribe certain drugs. On the other hand, CRS with the message not to prescribe certain drugs sometimes positively influenced the prescribing behavior of GPs. (Effect was not significant, Cohen’s d not calculated)	Not specific to asthma/COPD. Both groups served as a control group to one another. Authors report the study to be underpowered due to high inter doctor variation in prescribing behavior (Cluster effect). Training was provided.
15. Martens et al. [49] Netherlands. Follow-up of the above study.	20/48/-	Clustered RCT, Community setting. Duration: 1 year	25 GPs received reactive computer reminders on antibiotics and asthma/COPD prescriptions	28 GPs received (reactive) reminders on cholesterol prescriptions	Number of GPs (competent and willing) with CRS still functioning after 1 year. Number of GPs having technical problems or are unwilling. Number of reminders/GP/month/1000 enlisted patients. GP user satisfaction (satisfaction questionnaire). GP experience (content and extensiveness of CRS). Barriers and facilitators to implementation and use of CRS	(+) learning effect from the CRS. 9% of GPs dropped out after 1 year. A significant learning curve was found (P = 0.03) for the reminders on antibiotics, asthma and COPD. GPs were satisfied with the user-friendliness and the content of the different types of reminders, but less satisfied with certain specific technical performance issues of the system. Cohen’s d = N/A (Effect size not calculable due to insufficient data provided in report)	Not specific to asthma/COPD. Both groups served as a control to one another. GP’s were trained.
16. Kuilboer et al. [50] Netherlands.	32/40/156,772 (study patients (children and adults) either had chronic bronchitis, emphysema, other chronic pulmonary diseases, asthma or COPD) (I = 20, C = 20 General practitioners)	RCT, Community and academic Duration: 10 months	Asthma Critic used for monitoring and treatment of patients with asthma and COPD by Dutch general practitioners in daily practice. The asthma critic was a computer software support program that presented a patient specific comment to the physician based on the current clinical situation.	Usual care	Average number of contacts, FEV1 (force expiratory volume) and peak flow measurements per asthma/COPD patient per practice, and the average number of antihistamine, cromogylate, deptropine, and oral bronchodilator prescriptions per asthma/COPD patient per practice.	(+) effect. Statistically significant increase in contact frequency with the patient (P = 0.034), peak flow measurement, FEV_1_ measurements in 12-39 years age group (P = 0.02). Significant decrease in cromogylate prescriptions in the age group of 12-39 years, (P = 0.03). Non-significant decrease in deptropine, antihistamines, oral bronchodilators. (Effect size not calculable due to insufficient data provided in report).	The study focused on change in physicians’ behavior. Training was provided to the general practitioners.
17. Poels et al. [51] Netherlands	1 medical centre, several private practices/78/774 paper case descriptions. (10 case descriptions per GP).	Simulated cluster- RCT Community. Duration = 10 months	Expert support system for the interpretation of spirometry tests to help GPs’ in the diagnosis of chronic respiratory diseases. The expert system provided interpretation in the form of flow volume curve, graphical interpretation and textual interpretative notes of spirometry results to intervention GPs.	GPs in the control group simply received the spirometry test results, and the flow–volume and volume–time curves.	**Primary:** Difference between the percentage agreement of the cases’ diagnoses between GPs and expert panel judgment before and after interpretation of spirometry	No Effect. There were no differences between the computerized expert support and control groups in the agreement between GPs and expert panel on diagnosis of COPD, asthma and absence of respiratory disease. A higher rate of additional diagnostic tests was observed in the expert support group. (Effect was not significant, Cohen’s d not calculated)	This was a simulated study- no real patients involved. Training was provided.
					**Secondary:** Impact of the expert system intervention on the GPs decision-making processes through six measures: additional diagnostic test rates; width of differential diagnosis; certainty of diagnosis; estimated severity of disease; referral rate; and medication or non-medication changes.		
18. Poels et al. [52] The Netherlands	44/-/2098 (I = 15. C = 15. Chest physician = 14)	Cluster-RCT Duration: not mentioned.	Two interventions: GPs received spirometry interpretation support by either a chest physician (who had standard spirometry software) or expert spirometry support software.	Usual care had standard spirometry software (i.e. no additional interpretation support).	**Primary:** A change of diagnosis after spirometry interpretation support. **Secondary:** referral rate, additional diagnostic tests, and disease management changes.	No effect. Differences in proportion of changed diagnoses were not statistically significant. There were no differences in secondary outcomes. (Effect was not significant, Cohen’s d not calculated)	Training was provided.
19. Frickton et al. [53], USA.	15/102/59,147. (Patients with medically complex conditions like xerostomia, diabetes mellitus, COPD, congestive heart failure).	RCT with three arms (provider activation, patient activation and control group), Community setting. Duration = 2 years	Two CDS approaches. In one group, dentists and hygienists received alerts in the EDRs (electronic dental records) when patients scheduled for dental appointments had one of the targeted medical conditions. In second group, in addition to the above, patients with upcoming dental appointments who had one of the targeted medical conditions received a notification from HPDG (health partners dental group) before the visit, encouraging them to discuss it with his or her dental care provider at the appointment.	Patients in the control group received usual care. Neither the patients nor the provider’s, received alerts about a patient’s medical status or personalized care guidelines.	**Primary:** Total use-the overall frequency with which providers accessed the guidelines web site via the EDR for any patient. Targeted use—the proportion of providers who accessed the care guidelines in general and for targeted patients at the point of care. Ongoing use—the proportion of providers who continued to access the web-based guidelines through-out the study period.	(+) effect. Participants in the provider and patient activation groups increased their use of the system during the first six months. Provider activation was more effective than was patient activation. (P < 0.05). However, it was not sustainable, and by the end of the study, the rate of use had returned to baseline levels despite participants’ continued receipt of electronic alerts. (Effect expressed as Odds Ratio for web use for provider group in first six months = 4.4 (95% C.I = 1.6-12.1) and 6-12 months after implementation compared to controls = 1.7 (95% C.I = 0.1-2.9). For provider + patient activation group, effect expressed as Odds Ratio for web use in first six months =2.1 (95% CI, 0-9-4.8) and 6-12 months after implementation compared to controls = 1.4 (95% CI 0.5-3.5).	Dental clinic based. Study was not specific to asthma/COPD patients.

*Cohen’s d values calculated only for significant primary outcomes. Where primary data were not available, formulae to use P values/frequency tables to estimate d were used based on the Campbell Collaboration free online effect size calculator (available online at* http://www.campbellcollaboration.org/escalc/html/EffectSizeCalculator-SMD2.php *, accessed 9th September 2014). It must be noted that these effect sizes are estimates only.*

**Table 3:** **Quality assessment of the included randomized controlled trials**

Citation	Random sequence generation	Allocation concealment	Blinding of participants	Blinding of personnel	Blinding of outcome assessment	Incomplete outcome data	Selective reporting	Funding bias	Overall bias
1. Carroll et al. [35]	Low	Unclear	High	High	High	Low	Low	Low	High
2. Hashimoto et al. [36]	Low	Low	Unclear	Unclear	Low	Low	Low	Low	Low
3. Van der Meer et al. [37]	Low	Low	High	High	High	Low	Low	Low	Low
4. Van der Meer et al. [38]	Low	Low	High	High	High	Low	Low	Low	Low
5. Taylor et al. [39]	Unclear	Low	Low	Unclear	Low	Low	Low	Low	Low
6. Fiks et al. [40]	Low	Unclear	High	High	High	Low	Low	Low	High
7. Bell et al. [41]	Low	Unclear	High	High	High	Low	Low	Low	Moderate
8. Rassmusen et al. [42]	Low	High	High	High	High	High	High	High	High
9. Dexheimer et al. [43]	Unclear	High	High	Low	Unclear	Low	Low	Low	Moderate
10. Smith et al. [44]	Low	Low	Low	Low	Low	Low	Low	Low	Low
11. Kattan et al. [45]	Low	Low	High	High	Unclear	Low	Low	Low	Low
12. Tierney et al. [46]	Low	Low	Unclear	Low	High	High	Low	Low	Low
13. Martens et al. [47]	Low	High	Low	Unclear	Unclear	High	High	High	High
14. Martens et al. [48]	Low	High	Low	Unclear	Low	Low	Low	High	Unclear
15. Martens et al. [49]	Low	High	Low	High	Unclear	Low	Low	High	High
16. Kuilboer et al. [50]	Low	Low	Unclear	Low	Low	Low	Low	Unclear	Low
17. Poels et al. [51]	Low	Unclear	Low	Low	Low	Low	Low	Low	Low
18. Poels et al. [52]	Low	Unclear	High	Low	Low	Low	Low	Low	Low
19. Frickton et al. [53]	Low	Low	Low	High	Low	Low	Low	Low	Low

### Assessment of intervention effects

#### Type of CCDSS intervention provided

Trials were organized into three categories based on the type of CCDSS intervention provided (Table 4):

**Table 4:** **Type of CCDSS and its effectiveness**

Citation	CCDSS setting and format	CCDSS user	Type of CCDSS	Effect of CCDSS			
			Diagnostic/Drug therapy management only/Multifaceted CCDSS	Health care process outcomes (recommended preventative care, clinical study ordered, treatment ordered)	Clinical outcomes (morbidity, mortality, HRQOL, hospitalization, adverse events)	User workload and Efficiency outcomes (user knowledge, clinician workload, efficiency)	Relationship centered outcomes (patient satisfaction)/Economic outcomes (cost and cost effectiveness)/Use and implementation (health care provider acceptance, satisfaction, use and implementation)
1. Caroll et al. [35], USA.	Community/Integrated with the EMR	Practitioners	**Diagnostic CCDSS:** Clinician prompted to make an asthma diagnosis based on the results of a pre-screening questionnaire		**Primary outcome:** Significantly more children diagnosed with asthma (+ effect)		
2. Hashimoto et al. [36], Netherlands.	Hospital/Stand alone (Internet based)	Patients	**Drug therapy management based CCDSS:** Corticosteroid treatment decision support for the patients based on symptoms, lung function and exhaled NO (nitric oxide)	**Primary outcome:** Significant decrease in corticosteroid consumption in patients with steroid dependent asthma (+ effect)	No difference in asthma control, quality of life, FEV1, exacerbations, hospitalizations between groups. (+ effect)		No difference in patient satisfaction between groups
3. Van der Meer et al. [37], Netherlands.	Community/Stand-alone (Internet based)	Patients	**Multifaceted CCDSS:** Weekly asthma monitoring and advice, online and group education and remote web communications	Patients’ asthma knowledge, inhaler technique improved. Medication changes occurred more often. Health care provider contacts were fewer. (+ but modest effect)	**Primary outcome:** Asthma related quality of life improved. **Secondary**: Asthma control, lung function improved, symptom-free days increased, exacerbations did not differ between groups. (+ but modest effect)		
4. Van der Meer et al. [38], Netherlands.	Community/Stand-alone (Internet based)	Patients	**Multifaceted CCDSS:** Weekly asthma monitoring and self-management advice.	**Secondary:** Significant increase in the corticosteroid dose in patients with uncontrolled asthma, but not in patients with well or partly controlled asthma. (+ effect). Adherence to ACQ monitoring gradually declined in the first month to the seventh month and then remained stable. No difference in dose of corticosteroids or LABA or LRTA after 12 months	**Primary outcome:** Significant improvement in asthma control in patients with partly and uncontrolled asthma. (+ effect)		
5. Taylor et al. [39], Australia.	Hospital/Integrated	Practitioners (ED doctors)	**Multifaceted CDSS:** The system integrated asthma management pathways based on current guidelines into clinical and discharge documentation. Including triage and registration, clinical documentation, treatment orders, order entry and discharge documentation.			**Primary outcome:** Significantly higher rate of asthma documentation. **Secondary outcome:** No significant difference in consultation time. (+ effect)	
6. Fiks et al. [40], USA.	Hospital/Integrated into EHR	Practitioners	**Multifaceted CCDSS:** EHR based clinical alerts for influenza vaccine	**Primary outcome:** Increased Influenza vaccination rates. (+ effect, but not significant)			
7. Bell et al. [41], USA.	Hospital/Integrated into EHR	Practitioners	**Multifaceted CCDSS:** EHR based CDS alerts and reminders based on pediatric asthma management tool (PACT) which captured asthma symptom frequency, asthma severity, facilitated ordering of controller medications, spirometry and ACP (asthma action plan)	**Primary outcome:** Increase in the number of controller medication prescriptions, and up-to-date asthma action plan (ACP). (+ effect, but not significant). Increase in the use of spirometry in the intervention group (+ effect, but not significant)			
8. Rasmussen et al. [42], Denmark	Stand-alone (internet based)	Patients	**Multifaceted CCDSS:** Internet based asthma monitoring tool consisting of an asthma diary, action plan and a decision support for the physician	Significantly more patients using inhaled corticosteroids in the internet and specialist group (+ effect)	**Primary outcome:** Significant improvement in asthma symptoms, AQLQ, lung function, but no change in airway responsiveness (+ effect)		
9. Dexheimer et al. [43], USA.	Hospital/Integrated	Practitioners (ED physician)	**Multifaceted CCDSS:** Computerized detection system screened and identified patients with asthma exacerbation and a guideline based management protocol	**Secondary outcome:** No difference in asthma education charted, medication prescribed, follow-up appointment scheduled (No effect)	No difference in admission rate or ED length of stay (no effect)	**Primary outcome:** No significant difference in the time taken to make a ED disposition decision (no effect)	
10. Smith et al. [44], UK.	Community/Integrated (with the EHR)	Practitioners	**Multifaceted CCDSS:** EHR based alerts to flag the at-risk status of patients to improve patient access and opportunistic management	Relative increase in LABA usage and decrease in nebulized B-agonists (+ effect)	**Primary outcome:** No significant difference in the number of people experiencing exacerbations. Relative reduction in people experiencing hospitalizations, accident and emergency attendances, out-of-hour contacts and other health care use. (+ effect, but not significant)		Cost –effectiveness outcome: Adjusted mean health care (NHS) cost lower among intervention practices compared to control practices (+ effect)
11. Kattan et al. [45], USA.	Community/Stand-alone	Practitioners	**Drug therapy management based CCDSS:** Computer generated letter recommending change in controller medications based on NAEPP guidelines	**Primary outcome:** Significant increase in scheduled visits leading to stepping up of asthma medications (+ effect)	Significant decrease in ED visits. No difference in maximum number of symptom days and school days missed, decrease in the number of days with activity limitation.		Intervention-reduced asthma related cost to the health services and was cost-effective. (+ effect)
12. Tierney et al. [46], USA.	Hospital/Integrated	Practitioners (Physicians and pharmacists)	**Multifaceted CCDSS:** Care suggestions focusing on immunization, prescription and smoking advice	**Primary outcome:** No difference in the adherence to guideline-based care suggestions measured as the number of tests and treatment ordered (No effect)	No effect on quality of life, clinical symptoms, medication adherence and compliance, ED visits or hospitalizations (No effect)		Significantly higher health care costs in the group receiving only physician intervention. Physicians attitude towards guidelines was mixed
13. Martens et al. [47], Netherlands.	Community/Integrated	Practitioners (GPs)	**Drug therapy management based CCDSS:** Guideline based reminders when prescribing antibiotics, asthma/COPD and cholesterol prescriptions	**Primary outcome:** Reductions in the number of prescriptions according to the guidelines (+ effect, but not significant)			Providers perceived the CRS as stable and user friendly (+ effect, but not significant)
14. Martens et al. [48], Netherlands.	Community/Integrated	Practitioners (GPs)	**Drug therapy management based CCDSS:** Guideline based reminders when prescribing antibiotics, asthma/COPD and cholesterol prescriptions	**Primary outcome:** Clinically meaningful results seen in not prescribing certain drugs in the intervention group (+ effect, but not significant)			
15. Martens et al. [49], Netherlands.	Community/Integrated	Practitioners (GPs)	**Drug therapy management based CCDSS:** Guideline based reminders when prescribing antibiotics, asthma/COPD and cholesterol prescriptions			Significant learning curve was found (shows improvement in user knowledge) (+ effect)	**Primary outcome**: Provider use: Only 9% drop-out rate (because of technical problems requiring multiple updates) (+but not significant effect) Provider satisfaction: Positive attitude to the content of the reminders and satisfied with the user friendliness
16. Kuilboer et al. [50], Netherlands.	Community/Integrated	Practitioners (General practitioners)	**Multifaceted CCDSS:** Asthma critic evaluates whether the patient has asthma or COPD, reviews the physicians treatment, and generates feedback	**Primary outcome:** Significant increase in the average number of contacts. Significant decrease in the average number of cromogylate prescriptions. No statistically significant change in the antihistamines, deptropine, and oral bronchodilator prescriptions per asthma/COPD patient per practice (+ effect)	Significant increase in FEV1 (forced expiratory volume), and peak-flow measurements per asthma/COPD patient per practice (+ effect)		
17. Poels et al. [51], Netherlands.	Community/Stand-alone (spirometry expert system)	Practitioners (GPs)	**Multifaceted CCDSS:** Presentation of data for diagnosis and management of chronic airway disease	**Primary outcome:** No difference in between the two groups (Spirometry expert system and sham information) in the diagnosis of COPD, asthma and absence of respiratory disease or in medication changes. Secondary: Slightly more additional diagnostic tests in the expert group (No effect)			
18. Poels et al. [52], Netherlands.	Community/Integrated? (not clear)	Practitioners (GPs)	**Multifaceted CCDSS:** Spirometry expert support for change in diagnosis and management	**Primary outcome**: No differences in the proportion of changed diagnosis between the three groups (spirometry expert system, chest physician and usual care). Also no difference between the groups in referral rate, additional diagnostic tests and medication changes (No effect)			
19. Frickton et al. [53], USA.	Community/Integrated (with the EDR)	Practitioners (Dentists) and patients	**Multifaceted CCDSS:** EDR (Electronic dental record) based alerts notifying the dentists of the presence of a medically complex condition in a patient with a link to modify dental care appropriately				**Primary outcome**: Significant increase in the frequency of dentists accessing guidelines (number of website hits and number of providers using the guideline). Only number of hits sustained after 6 months. After 9 months provider use returned to baseline levels (+ effect)

1. Diagnostic advice only;
2. Drug therapy management only; and
3. ‘Multi-faceted’ interventions comprising two or more different intervention components.

#### Effectiveness of CCDSS

Using classifications published in previous reviews pertaining to CCDSS [54], we assessed CCDSS effectiveness based on the following key outcomes:

1. Clinical outcomes: Morbidity, health related quality of life, hospitalizations and mortality. [e.g. asthma symptoms (measured using symptom diary), asthma control (Asthma control Questionnaire-ACQ), lung function (Piko-1 device, peak flow meter), health-related quality of life (measured using HRQOL) and adverse events (leading to unscheduled doctors visit or hospitalization)];
2. Healthcare process measures: Recommended preventive care services ordered or completed (e.g. influenza vaccination), recommended clinical study ordered or completed (including spirometry), recommended treatment ordered or completed (including rescue medication prescriptions and antibiotic prescriptions)];
3. User workload and efficiency outcomes: Effect on user knowledge, number of patients seen per unit time, clinician workload, and efficiency;
4. Relationship-centered outcomes: Patient satisfaction surveys;
5. Economic outcomes: Cost and cost effectiveness of the CCDSS used; and
6. Use and Implementation outcomes: Health care provider acceptance, health care provider satisfaction, and health care provider use and implementation.

A CCDSS was considered effective if it produced a statistically significant (p <0.05) improvement in the primary outcome or improvement in ≥50% of multiple relevant pre-specified outcomes. If the authors did not designate a primary outcome, we considered the outcome used to calculate the trial’s sample size to be primary. Studies that included multiple intervention arms were considered effective if any of the CCDSS based treatment arms was evaluated as effective.

Although we had intended to conduct meta-analyses, this was abandoned owing to the marked heterogeneity in participants, clinical settings, interventions, and the outcomes measured in the included studies. However, effect sizes (Cohen’s d value) of the significant primary outcomes were calculated wherever possible.

## Results

**Figure 1:**
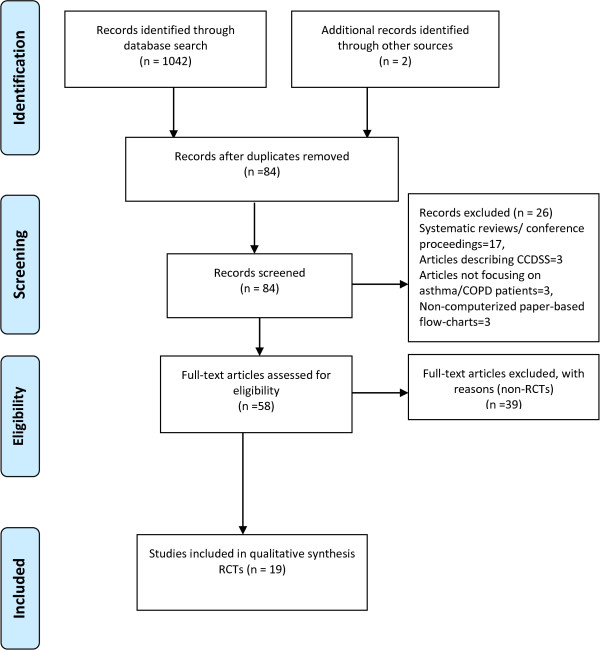
**PRISMA flow diagram of the included and excluded studies** [55] **.**

The PRISMA guidelines for conducting/reporting systematic reviews were consulted and a completed checklist is attached as Additional file 1. We screened 1042 abstracts, identified 173 full-text potentially relevant articles and included 19 articles representing 16 RCTs in the review (Figure 1).

Studies were predominantly conducted in the Netherlands (n = 9) and the USA (n = 7) with one study conducted in each of the following countries: Australia, United Kingdom and Denmark.

Eleven studies evaluated asthma care [35–45], 5 studies involved patients with asthma and COPD [46–50], and 3 studies focused on people with medically complex conditions including COPD [51–53]. There were no studies conducted exclusively on people with COPD (Table 2).

### Study quality

A summary of the study quality of the included studies is reported in Table 3. Of the 19 trials, 10 studies had a low risk of bias [36–39, 44–46, 51–53]. Eleven studies described an appropriate method of sequence generation [35–38, 44–46, 50–53], 9 studies reported adequate concealment of allocation [36–39, 42, 44, 46, 50, 53], and 13 studies showed either no differences in baseline characteristics between study groups or performed appropriate adjustments [35–37, 39, 40, 42–46, 50, 51, 53]. Eleven studies used objective outcomes or blinding of outcome assessments [36, 39, 40, 43–45, 48, 50–53], and 11 studies achieved a ≥90% follow-up rate for their unit of analysis [35–39, 44–46, 48–50].

### CCDSS and study characteristics

Table 2 describes the CCDSS design and implementation characteristics. The majority of interventions (68%) (13/19) were embedded in an existing electronic medical record (EMR) or with the computerized physician order entry (CPOE) systems [35, 39–41, 43, 44, 46–52]. Thirty-one percent (6 studies) had a stand-alone system, of which four were internet based [36–38, 42] and in the other 2 studies CCDSS intervention were administered to practitioners by the study researchers [45, 52]. Five of the six studies with the stand-alone CCDS system showed positive impact. Sixty-three percent (12 studies) automatically pre-populated the EMR data [35–41, 43, 44, 46, 50, 53], 26% (5 studies) relied on practitioners to manually enter the data [42, 47–49, 52], 16% (3 studies) relied on research staff [44, 45, 51] and 21% (4 studies) relied on patients as well for data inputs [36–38, 42]. Forty-seven percent (9 studies) compared a computerized clinical decision support system directly with usual care [35–38, 40, 43, 44, 50, 52].

Advice at the point-of-care was provided in 14 trials [35, 39–41, 43, 44, 46–53] and via the internet in 4 trials [36–38, 42]. Advice in the form of a computer-generated letter recommending changes to the treatment was provided to the practitioners in one study [45]. Advice was provided only to the physicians in 68% (13 trials) [35, 39–41, 43, 44, 46–52], while only one trial involved provision of advice to other healthcare practitioners (pharmacists) in addition to physicians [46]. In 26% (5 studies) patients were directly advised in addition to practitioners [36–38, 42, 53]. Thirteen studies provided explicit training in use of the CCDSS to healthcare practitioners [39, 41–52], while patients were trained to use the internet based CCDSS in 3 trials [36–38]. The CCDSS user interface characteristics were described in only 42% (8) of the trials [35, 38, 39, 43, 48–50, 53].

Studies included a highly varied number of healthcare practitioners, patients and health services.

Since CCDSS is primarily focused on altering provider behavior, the unit of randomization in most CCDSS studies was the provider. Thirty-seven percent (7) of the studies reviewed were randomized at the provider, practice or community level [39, 45, 46, 50–53], while 31% (6) used cluster randomization either between clinics or groups of providers that worked closely together [40, 41, 44, 47–49]. Thirty-one percent (6 studies) were randomized at the patient level [35–38, 42, 43].

In all studies that included patient level randomization there was potential for contamination given a single provider could care for both intervention and control arm patients. The principal summary measures used to compare effects between intervention and control groups varied and included: proportions [35, 39, 41, 45]; difference in medians [36, 38, 43, 50]; difference in means [46, 48]; relative risk [37] and odds ratios [40, 42, 44, 51–53].

Three trials did not clearly report their source of funding [41, 42, 53]. Of the remaining, 9 trials were publicly funded [36, 37, 41, 43, 45, 46, 51–53], 5 trials were conducted with only private funds [40, 44, 47–49], 1 trial was conducted with a combination of private and public funding [42], while another trial did not receive any funding [35]. Five trials declared that at least one author was involved in the development of the CCDS system [37, 38, 47–49], while the remaining trials did not indicate at all if the authors were independent of development.

### Type of CCDSS interventions

The included studies utilized CCDSS for a variety of purposes, and were categorized in to three main categories, such as those focusing on screening/diagnosis, drug therapy management, and multifaceted interventions which involved various aspects of disease management along with self-management advice (Table 4).

There was only one study, conducted by Caroll et al. that used the CCDSS (Child Health Improvement through Computer Automation-CHICA system) for the purpose of diagnosing pediatric asthma [35]. Five studies (26%) used the CCDSS for drug therapy management [36, 45, 47–49], and thirteen used a multi-faceted form of CCDSS [37–44, 46, 50–53].

The studies evaluating the CCDSS focusing on drug therapy management included the study by Hashimoto et al. which utilized an Internet based treatment decision support system to guide people with severe asthma in tapering the dose of oral corticosteroids depending on their asthma control [36]. The study by Kattan et al. involved provision of a computer-generated letters to the treating physician summarizing the appropriate treatment recommendations based on the child’s asthma symptoms, health service and medication use [45]. The three studies conducted by Martens et al. also involved a CCDSS in the form of reactive computer reminders (CRS) to improve drug prescribing in general practice [47–49].

The remaining 13 studies (68%) evaluated multifaceted forms of CCDSS [37–44, 46, 50–53]. These CCDSSs ranged from simple activation of electronic alerts to identify people at risk of an asthma exacerbation [44], or prompts to alert the physician to modify treatment in people with medically complex conditions at the point of care [53], to more complex forms of CCDSS interventions involving a series of care suggestions on drug therapy and disease management [46]. Internet-based multifaceted CCDSS interventions were evaluated in three trials which focused on the self-management of asthma [37, 38, 42]. These studies utilized online self-management programs which involved weekly online asthma control monitoring and feedback in the form of treatment advice by a specialized asthma nurse [37, 38], or by the patients’ physician [42]. Another type of multifaceted CCDS interventions evaluated in two other studies were in the form of EHR-based clinical alerts either to improve influenza vaccination in children with asthma [40], or to improve overall asthma care in these children [41]. The multifaceted CCDSS evaluated by Kuilboer et al. was a critiquing system integrated with the general practitioners’ electronic medical records which reviewed physicians’ treatment decisions and generated feedback [50]. Another two studies evaluated the impact of an expert spirometry system on the physician’s decision making during asthma diagnosis [51] and during management [52]. The remaining two trials also tested the effects of another multifaceted CCDSS on the clinician’s performance in the form of an electronic interface system to manage asthma patients in ED [39] and in the form of an automated asthma detection system used to identify and manage people at risk of asthma exacerbation in the emergency department [43].

### CCDSS effectiveness

There was marked variability in the outcomes reported. Therefore we assessed the effectiveness of the CCDSS on the primary outcomes measured. In majority of the trials reviewed, the primary outcomes assessed were health care process measures, clinical outcomes, user work load and efficiency, and use and implementation outcomes. Relationship-centered outcomes and economic outcomes were measured by few trials, but only as secondary outcomes. Fourteen trials (74%) showed positive effect from the use of CCDSS on the primary outcome measured, of these 9/19 (47.3%) showed a significantly positive effect [35, 36, 38, 39, 41, 42, 45, 50, 53].

### Clinical outcomes

The different clinical outcomes reported in the studies included asthma symptoms [42], asthma/COPD symptoms [46] asthma control (ACQ) [36–38], Health related Quality of life [36, 37, 42, 46], frequency of health care utilizations including hospitalizations [36, 44], admission rate and ED length of stay [43], frequency of exacerbations [36, 37, 44], lung function (FEV1) in asthma patients [36, 37, 42], exhaled nitric oxide [36], symptom free days [37], airway hyper responsiveness [42], number of ED visits [45, 46] number of school days missed [45], medication adherence [46], FEV1 and peak flow measurements in asthma/COPD patients [50].

Five of the nineteen trials assessed clinical outcomes as the primary outcome measure [35, 37, 38, 42, 44], of which three showed clinically significant improvements [35, 38, 42], one showed a positive but modest improvement in the asthma related quality of life [37] and another one did not show any effect on the number of people experiencing an exacerbation from the use of EHR embedded asthma risk alerts [44].

Significant improvement was found in the rate of diagnosis of asthma in children by implementation of a parent survey linked to physician prompts using computer decision support system called the CHICA system [35]. Significant improvement was also found in asthma control measured weekly using the asthma control questionnaire (ACQ) [38], in asthma symptoms using an electronic diary to record symptoms daily and in the asthma quality of life measured using asthma quality of life questionnaire (AQLQ) [42]. The effect sizes (Cohen’s d) calculated for the studies showing significant improvement in the primary clinical outcomes ranged from 0.24 to 0.94, with three studies showing a reasonably large effect size [37, 38, 42].

### Health care process outcomes

The different health care process measures that were assessed in the reviewed trials included change in the consumption of oral corticosteroid [36], change in the dose of inhaled corticosteroid [38, 42], change in patients’ asthma knowledge [37], change in inhaler technique [37], change in medication adherence [37], medication changes [37, 38, 44], adherence to the use of ACQ [38], rate of vaccination [40], number of corticosteroid prescription ordered [41], provision of asthma action plan [41], spirometry ordered [41], rate of asthma documentation by ED doctors [43], scheduled physician visits leading to change in medication dose [45, 50], physicians adherence to guidelines [46], change in the number of prescriptions [47, 48, 50], change in the diagnostic ability of the general practitioner [51, 52], diagnostic tests ordered [51, 52] and the rate of referral [52].

Ten trials assessed health care process measures as the primary outcome [36, 40, 41, 45–48, 50–52], of which four showed significant improvement in these outcomes. Significant improvement was seen in process outcomes like cumulative sparing of prednisone dose adjusted weekly according to the internet based CDSS [36], percentage of children given at least one prescription of corticosteroid [41], percentage of visits to the physician leading to medication step up of asthma medication [45] and in the number of contacts with the patients’ physician [50]. The effect size calculated for the two studies [36, 45] with significantly positive improvement was however poor. Three trials showed a positive but modest effect of which one showed a modest improvement in the rate of influenza vaccination by the use of EHR alerts [40]. The other 2 trials showed a modest improvement in the drug prescribing behavior of GPs from the use of the CRS reminders [47, 48]. The remaining 3 studies did not show any effect from the use of CCDSS on the primary health care process outcome assessed.

### User workload and efficiency outcomes

Workload and efficiency outcomes assessed in the trials included asthma documentation by emergency department (ED) doctors [39], consultation time [39], time for disposition decision in the ED [43], and user knowledge [49]. These outcomes were assessed as the primary outcome by only two trials [39, 43], of which one trial showed significant improvement in the rate of asthma documentation by the ED doctors in the management of acute asthma [39]. The size of the effect calculated for this trial was relatively large (Cohen’s d =0.78). However the other trial did not show any effect from the use of CCDSS on the time taken by the ED physicians to make a disposition decision [43].

### Use and implementation outcomes

The outcomes assessed under this category were physicians attitude to guidelines [46], user friendliness [48, 49], provider satisfaction [49], and the rate of accessing guidelines [53]. Two trials assessed these outcomes as the primary outcome, of which one showed a significant improvement in the rate of use of guidelines by dentists in the management of people with chronic diseases including COPD [53]. The study showed that the use of CCDSS increased the number of times the dentists accessed the guidelines. The other trial also showed a positive but modest effect in the use of CRS (reactive computer reminders) by general practitioners, not to prescribe certain drugs [49].

### Other outcomes

Outcomes such as patient satisfaction with CCDSS use was measured as a secondary outcome by two trials and found no difference in patient satisfaction between the intervention and the control group patients [36, 46]. Measures such as health care provider satisfaction were also assessed as secondary outcomes. Of the three studies that measured these outcomes [46, 47, 49], two found that the provider perceived the CCDSS as user friendly. Only one trial measured cost of the intervention and found that the patients in the group receiving the CCDSS intervention had significantly elevated total health care charges [46]. Two other trials measured the cost-effectiveness of the CCDSS used and both found that its use was more cost effective than usual asthma care [44, 45].

## Discussion

This is the first comprehensive review of CCDSS in the care of patients with chronic respiratory diseases, asthma and COPD. The review focused only on studies conducted in primary care as the bulk of the management of these chronic diseases happens in primary care. The review found that the use of CCDSS can have a positive impact on the diagnosis and management of asthma and COPD in primary care. Overall 74% of the studies reviewed showed improvement in the primary outcomes. Although there is literature available on the use of CCDSS in patients with asthma, there is very little literature on its use in the management of people with COPD.

The review also found that 83% (5/6) of the studies that utilized CCDSS with a stand-alone design showed positive outcomes as compared to studies testing CCDSS which were integrated with the EHR or the EMR systems (38%) (5/13). This indicates that systems presenting advice within electronic health records or order entry systems were much less likely to improve care or outcomes than stand-alone programs. It has been found that when integration of alerts within an institution’s electronic health records becomes possible and more alerts are added, practitioners might become overwhelmed and begin to ignore the prompts. This “alert fatigue” phenomenon [56] could be responsible for limiting behavior change. Studies estimate that as many as 96% of alerts are over ridden [57–59] and suggest that the threshold for alerting is too low (that is, alerts are sensitive but not specific). Systems requiring the practitioner to give a reason for over-riding advice were more likely to succeed than systems missing this feature [60].

Four of the five studies evaluating CCDSS with a stand-alone design, were Internet-based interventions targeting both physicians and patients. All the four studies showed that CCDSSs which targeted the patients as well as the physicians were effective in improving outcomes. The findings are consistent with other previous reviews of CCDSS for chronic disease management in primary care [33, 61]. A key feature of these interventions was the active incorporation of a patient self-management component for use outside of the clinical encounter. The CCDSS interventions included in the studies involved regular monitoring and feed-back along with patient education and follow-up. These results confirm the value of collaborative care in chronic respiratory disease management.

Also CCDSS interventions consisting of multiple components such as reminders and education were associated with greater improvement in outcomes than single-target interventions with fewer components. This is also reflected in other reviews evaluating the effectiveness of such multi-component CCDSSs engaging patients in the management of other chronic conditions like diabetes [33] and osteoporosis [62]. Given the advent of personal health records, patient portals, and mobile applications aimed at better engaging patients, the findings suggest that there is a need to consider multiple components and targets in the development of any future interventions.

Of the outcomes measured many of the included studies (53%) often focused on measuring the effectiveness of CCDSSs on the health care process outcomes and the evidence demonstrating the effects of CCDSSs on patient outcomes, user workload and efficiency and economic outcomes remains surprisingly low. This is comparable to other recent CCDSS reviews which also report on the paucity of well-designed studies evaluating the effects of CCDSS on patient related outcomes [32, 63]. This may have occurred owing to under powering, since most of the studies may not have had large enough sample sizes to detect such outcomes. Similarly many of these studies were not conducted over longer time frames. Both the sampling and time issues were possibly due to the relative difficulty of implementing randomized, controlled trials in real clinical settings [54]. Since clinical decision support has a primary function aimed at providing information to the provider at the point of decision making and intervention, outcomes which measure process or provider behavior are often used as a proxy for patient outcomes [61]. Although analysis of process outcomes has a merit as an interim platform to justify the continuing role of CCDSS in clinical care, more research is needed on evaluating the effectiveness of CCDSS on patient outcomes in order to adequately understand the usefulness of CCDSS in clinical setting. Nevertheless, 60% of the 5 studies measuring clinical outcomes showed significantly positive impact on these outcomes as compared to 40% of the 10 studies showing significant improvement in health care process outcomes. This implies that the implementation of CCDSS for asthma/COPD care seems promising in improving clinical outcomes. The most commonly reported clinical outcomes were asthma control and asthma quality of life.

CCDSSs may represent a cost-effective way of improving chronic respiratory disease outcomes in primary care. However, the review found that the economic effects of these systems could not be readily assessed based on the available data. The costs of design, local implementation, ongoing maintenance, and user support can be high, and may be further elevated by the unique nature of chronic respiratory care. This warrants cost-effectiveness analyses, but only two trials reported such data and little cost data of any kind was available across studies. Almost all of the studies discussed the need for more research utilizing cost-effectiveness outcomes to better assess the long term effectiveness of CCDSS.

The review also found that there were no studies that demonstrated a negative finding (patient harm or deterioration related to the intervention). This could be because the studies did not actively collect any data on harm assessment of the CCDSS used. Prospective data on the possible harms of CCDSSs are needed to facilitate informed adoption decisions. Based on the available evidence it is hard to draw conclusions about the potential negative effect of implementing decision-support tools, which is necessary to truly fulfil the goal of evaluating these interventions and to better address implementation challenges [64].

### Strengths and limitations

The review has several important strengths. This is the first review evaluating the role of CCDSS in the management of chronic respiratory diseases, asthma and COPD in primary care. We particularly excluded studies regarding in-patient hospital based CCDSSs as we intended to focus its effectiveness in primary and community health care, given that only a small proportion of people with, asthma for example, are managed in the hospital setting. The search strategy of our study was extensive and thorough, and covered a large number (eight) of relevant databases to identify potentially relevant studies. The other strength is we based our review on the strongest studies available, RCTs. Also to reduce the risk of selection bias and incorrect categorization all the included articles were analyzed and critically examined by three reviewers independently.

There are a number of key limitations to this review. We excluded studies regarding in-patient hospital based CCDSSs as we intended to focus its effectiveness in primary and community health care. We included only English language studies conducted in the last 10 years as we wanted to document the recent advances in this area. Our analyses were limited to published reports of randomized controlled trials, so the possibility of publication bias or selective reporting must be acknowledged. The CCDSSs were grouped into categories based on clinical applications rather than on other aspects of CCDSS design. We were unable to conduct meta-analysis, given the substantial heterogeneity in the type of CCDSSs and the outcomes evaluated, however we calculated the effect sizes of the primary outcomes for easier comparison of the study effects. Finally, we summarized only randomized controlled trials which might have resulted in less information about issues related to CCDSS implementation, effect on workflow, and factors affecting usability.

## Conclusion

In summary, the review demonstrates that CCDSS can improve chronic disease management processes and clinical outcomes in patients with asthma and COPD, but data showing its effect on use and implementation and economic outcomes were sparse. The review also found that although there are a growing number of RCTs that assessed a wide variety of CCDSSs designed to improve asthma management in primary care, there is very scant evidence of its use in the care of patients with COPD.

The mechanisms behind systems’ success or failure remain understudied, but non-integrated, multifaceted CCDSS providing advice to both practitioners and patients, and those requiring the practitioners to give explanations for over-riding advice might independently improve success.

Future trials with clear descriptions of system design, local context, implementation strategy, costs, adverse outcomes, user satisfaction, and impact on user workflow will better inform CCDSS development and decisions about local implementation.

## Electronic supplementary material

Additional file 1: **PRISMA checklist.** (DOC 61 KB)

## Abbreviations

COPD: Chronic Obstructive Pulmonary Disease
CCDSS: Computerized clinical decision support systems
EMR: Electronic medical records
EHR: Electronic health records
CRS: Computer reminder system
CPOE: Computerized physician order entry
EDR: Electronic dental records.
